# The contribution of hospital-acquired infections to the COVID-19 epidemic in England in the first half of 2020

**DOI:** 10.21203/rs.3.rs-1140332/v1

**Published:** 2022-03-03

**Authors:** Gwenan Knight, Thi Mui, James Stimson, Sebastian Funk, Yalda Jafari, Diane Pople, Stephanie Evans, Mo Yin, Colin S. Brown, Alex Bhattacharya, Russell Hope, Malcolm G. Semple, Jonathan M Read, Ben S Cooper, Julie V. Robotham

**Affiliations:** London School of Hygiene & Tropical Medicine; Julius Center for Health Sciences and Primary Care: Julius Centrum voor Gezondheidswetenschappen en Eerstelijns Geneeskunde; Public Health England; London School of Hygiene & Tropical Medicine; London School of Hygiene & Tropical Medicine; Public Health England; Public Health England; University of Oxford Nuffield Department of Medicine; Public Health England; Public Health England; Public Health England; University of Liverpool; Lancaster University; Univerity of Oxford Nuffield Department of Medicine; Public Health England

**Keywords:** COVID-19, SARS-CoV-2, nosocomial transmission, mathematical modelling

## Abstract

**Background:**

SARS-CoV-2 is known to transmit in hospital settings, but the contribution of infections acquired in hospitals to the epidemic at a national scale is unknown.

**Methods:**

We used comprehensive national English datasets to determine the number of COVID-19 patients with identified hospital-acquired infections (with symptom onset >7 days after admission and before discharge) in acute English hospitals up to August 2020. As patients may leave the hospital prior to detection of infection or have rapid symptom onset, we combined measures of the length of stay and the incubation period distribution to estimate how many hospital-acquired infections may have been missed. We used simulations to estimate the total number (identified and unidentified) of symptomatic hospital-acquired infections, as well as infections due to onward community transmission from missed hospital-acquired infections, to 31^st^ July 2020.

**Results:**

In our dataset of hospitalised COVID-19 patients in acute English hospitals with a recorded symptom onset date (n = 65,028), 7% were classified as hospital-acquired. We estimated that only 30% (range across weeks and 200 simulations: 20-41%) of symptomatic hospital-acquired infections would be identified, with up to 15% (mean, 95% range over 200 simulations: 14.1%-15.8%) of cases currently classified as community-acquired COVID-19 potentially linked to hospital transmission.

We estimated that 26,600 (25,900 to 27,700) individuals acquired a symptomatic SARS-CoV-2 infection in an acute Trust in England before 31st July 2020, resulting in 15,900 (15,200-16,400) or 20.1% (19.2%-20.7%) of all identified hospitalised COVID-19 cases.

**Conclusions:**

Transmission of SARS-CoV-2 to hospitalised patients likely caused approximately a fifth of identified cases of hospitalised COVID-19 in the “first wave” in England, but less than 1% of all infections in England. Using time to symptom onset from admission for inpatients as a detection method likely misses a substantial proportion (>60%) of hospital-acquired infections.

## Background

The SARS-CoV-2 pandemic is a global public health priority.(1) Based on experience with other highly pathogenic coronaviruses within-hospital transmission can occur and hospitals may play an important role in amplifying transmission.(2) Moreover, many patients acquiring SARS-CoV-2 in hospitals are at high risk for severe outcomes and subsequent mortality.(3) Quantifying hospital-acquired transmission of SARS-CoV-2 is thus important both for prioritising control efforts and for understanding the contribution of hospitals to sustaining the community epidemic.

SARS-CoV-2 transmission in healthcare settings has been reported in many countries.(3-6) As the precise time of infection is rarely known, establishing whether an infection is hospital-acquired remains a challenge. For SARS-CoV-2, hospital-acquired infections are usually defined by comparing the time of admission and subsequent symptom onset(7) or first positive test.(8) If the delay is much longer than the incubation time, then it is likely that an infection is hospital-acquired. Thus, the proportion of patients with a hospital-acquired SARS-CoV-2 infection will depend on the definition used, with uncertainty driven by the unobservable nature of infection and the incubation period distribution. Records for all hospitals in England, using testing data and definitions of hospital-acquired if first positive sample is taken more than 14 days from admission, indicate that 15% of detected SARS-CoV-2 infections in hospitalised patients could be attributed to hospital-acquired transmission (8) with analysis of data from single hospital facilities suggesting a similar level.(3,9)

In the absence of frequent universal testing of all inpatients, many hospital-acquired SARS-CoV-2 infections will not be identified by hospitals prior to discharge. Even with regular PCR testing of all inpatients regardless of symptoms we would expect to miss many infections because of short patient stays and potentially low PCR sensitivity 1-2 days after infection.(10)

**Figure 1:**
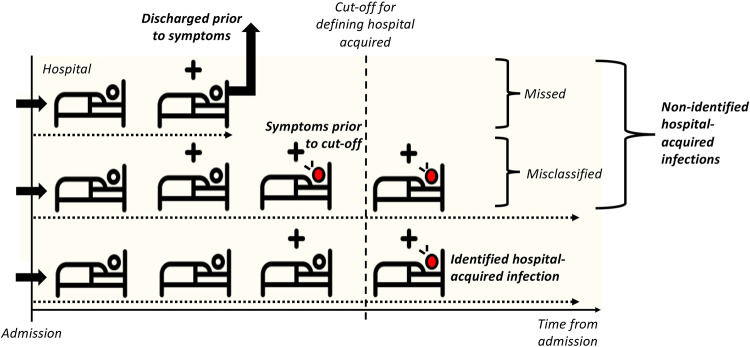
How might we underestimate hospital-acquired (HA) infections? With no asymptomatic screening in hospitals, detection of a hospital-acquired case relies on symptom onset prior to patient discharge. In the schematic a “+” above the bed denotes a hospital-acquired infection, and a red patient denotes one with symptoms. A patient with COVID-19 identified as being due to a hospital-acquired infection is one with symptom onset after a defined cut-off (e.g. >7 days from admission to symptom onset but prior to discharge, bottom row patient). Patients with unidentified hospital-acquired infections are those with a symptom onset after discharge (top row patient, “missed”) or those with symptom onset prior to the defined cut-off (middle row patient, “misclassified”). We focus on symptomatic infection: there will also be unidentified asymptomatic hospital-acquired infection which we do not include. We estimate that fewer than 1% of individuals with symptom onset >7 days from admission will have been infected in the community.

In the spring of 2020 in England, the majority of inpatient testing only occurred in those with symptoms, either on admission or during hospital stay.(11) Many patients who develop a symptomatic infection will do so after discharge (Figure 1) as hospital stays are typically shorter than the interval from infection to symptom onset (median length of stay = 2.4 days, standard deviation = 0.4 days, for non-COVID patients in England vs. incubation period average of 5.1 days (12)). Thus, there may be a considerable proportion of hospital-acquired infections that remained unidentified. Its magnitude and further transmission to the community has been difficult to quantify. Additionally, a substantial proportion of infected individuals never progress to be symptomatic.(13)

In this analysis, we used national, patient-level datasets of patients hospitalised with COVID-19 to estimate the contribution of hospital settings to the first wave of COVID-19 in acute Trusts in England. We estimated the proportion of symptomatic hospital-acquired infections that have not been identified as hospital-acquired and modelled onward transmission from these unidentified infections in the community. We hence quantified the likely contribution of symptomatic hospital-acquired infections to the first wave of SARS-CoV-2 infections in England.

## Methods

Our primary aim was to estimate the total number of symptomatic hospital-acquired SARS-CoV-2 infections in England from 1st January to 31st July 2020. For each identified symptomatic hospital-acquired infection, we estimated how many were unidentified. Our secondary aim was to estimate the contribution of these unidentified hospital-acquired infections to the community epidemic.

**Figure 2:**
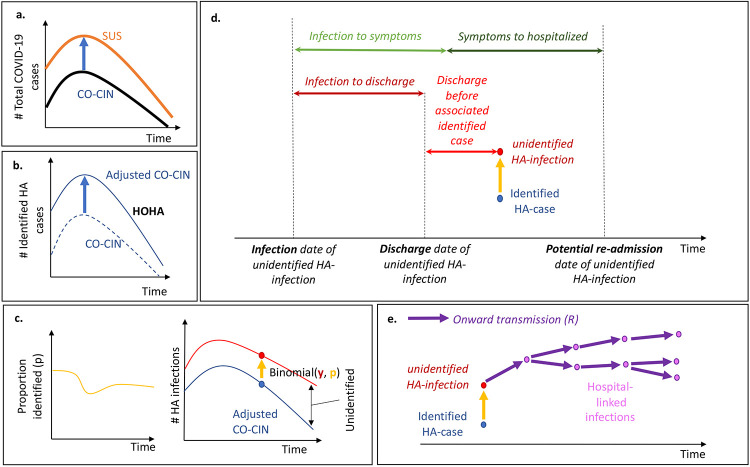
The analysis steps: (a) CO-CIN is inflated to match total COVID-19 hospitalised cases in SUS. (b) The same weekly adjustment is used to estimate the number of identified hospital-onset, hospital-acquired (HOHA) cases. (c) The length of stay for non-COVID-19 hospital patients and incubation period distribution is used to generate estimates of the proportion of hospital-acquired infections that would be identified (Figure 1). This proportion (*p*) is used to estimate how many unidentified hospital-acquired infections there would be for each identified hospital-onset hospital-acquired infection by assuming a Binomial distribution and calculating the number of “trials” or “unidentified” hospital-acquired infections there were. (d) The unidentified hospital-acquired infections with symptom onset after discharge (“missed”) may return to hospital as a COVID-19 case: the trajectory of their disease is calculated to determine their contribution to hospitalised cases. (e) These “missed” unidentified hospital-acquired infections are assumed to contribute to onward transmission in the community: here we capture four generations of transmission to estimate the number of hospital-linked infections and subsequent hospitalised cases under different *R* estimates.

All analyses were conducted in *R* version 4.0.3(14) with code available on Github.(15) The steps in the analysis are outlined in Figure 2.

### Data sources

The healthcare system in England is represented by the National Health System (NHS). NHS services are mainly provided by NHS Trusts, i.e., collections of hospitals (departments, buildings and facilities) that function as a single administrative unit. Acute medical care Trusts are defined as an NHS Trust with only acute hospitals (as opposed to Community or Mental Health facilities). In this study, we used two data sources on COVID-19 patients admitted to NHS Trusts (Supplementary 2). The first is the ISARIC4C UK COVID-19 Clinical Information Network (CO-CIN) study,(16) a national cohort of COVID-19 patients collected in 208 acute Trusts in England, Scotland, and Wales up to 3^rd^ December 2020, representing approximately two thirds of COVID-19 UK admissions during the first wave of SARS-CoV-2 infection. While not all NHS Trusts are represented in the data (as some have specialist roles that do not involve inpatient acute medical care), our CO-CIN extract comprised 208 of 223 acute medical care Trusts. (17,18) We included 126 Trusts in England and filtered the dataset for patients with a symptom onset before 1^st^ August 2020. CO-CIN recorded admission date, discharge date, and earliest date of symptom onset for patients. We excluded CO-CIN participants without a recorded admission and symptom onset date (Supplementary 2).

The second is the SUS dataset(19) which contains data on all patient admissions and discharges for all Trusts in England. The SUS data were linked with testing data (Second Generation Surveillance System (SGSS))(19) to derive length of stay distributions for non-COVID-19 patients and total COVID-19 hospital admissions by week and NHS Trust.

These two data sources have their respective strengths and limitations. The CO-CIN data include information on the date of symptom onset(20) but are only a subset, albeit the majority, of all hospitalised COVID-19 patients, while the linked SUS/SGSS data include all known hospitalised COVID-19 patients but lack information on symptom onset date. Symptom onset dates do not rely on knowledge of testing regimens which vary over time and between Trusts. To address these different issues, we decided to use SUS data to adjust CO-CIN information to account for enrolment variation between settings, giving a database combining the best features of both.

### Setting

Our baseline population is all acute English Trusts in CO-CIN. These are aggregated as a single “England” population for our main analysis. A sensitivity analysis modelled the individual acute Trust level prior to aggregation (Supplementary 12).

### Length of stay distribution

We used empirical length of stay (LoS) estimates for non-COVID-19 patient stays from SUS for each English acute Trust in CO-CIN for patients admitted each week (Supplementary 2). To get a LoS distribution for England, LoS estimates across all including Trusts were pooled by week. The average length of stay was between 1.5 and 2.5 days across this time period.

#### a. Identifying COVID-19 cases as infected in hospital

The number of identified hospital-acquired COVID-19 cases per day in each Trust was estimated by comparing the dates of symptom onset and hospital admission for each in-patient within CO-CIN. Our analysis used a 7 day cut-off: we defined an identified hospital-acquired infection as an inpatient with symptoms onset more than 7 days after admission (Table 1) aligned with English definitions and the ECDC definition for a Probable (8-14) and Definite (>14days) healthcare-associated COVID-19 case(7,21). As such, identified hospital-acquired infections are by definition symptomatic infections. In sensitivity analyses we explored cut-offs of 4 and 14 days.

#### b. Accounting for enrolment into CO-CIN

We accounted for the fact that only a subset of all hospitalised COVID-19 patients was enrolled in CO-CIN as follows: We calculated the proportion of COVID-19 patients recorded in SUS in a given week that were included in the corresponding CO-CIN data. We then weighted the weekly estimates of the number of identified hospital-acquired infections from the CO-CIN data using the inverse of these weekly proportions to obtain estimates of identified hospital-acquired COVID-19 cases corrected for under-reporting in CO-CIN (Supplementary 4). Our method assumes that there is no bias in enrolment of hospital- versus community-onset cases.

#### c. Proportion of hospital-acquired infections that are identified

Not all symptomatic infections with SARS-CoV-2 are identified (e.g., some individuals are infected with SARS-CoV-2 in hospital and subsequently have symptoms that are not confirmed to be COVID-19). All identified cases of COVID-19 with symptom onset in a hospital setting are classified as either hospital- or community-acquired. However, some are misclassified (e.g., those that are infected in hospital but have a symptom onset prior to the cut-off threshold for defining hospital-acquired cases). Our aim was to estimate both overlooked symptomatic SARS-CoV-2 infections that were not identified and that were misclassified (Figure 1, Table 1). We did not consider those who acquire infection but remain asymptomatic.

To calculate the proportion of symptomatic hospital-acquired infections that were identified as such, we calculated the probability that a patient with a hospital-acquired infection has a symptom onset that falls in the definition period, i.e., before discharge and after the cut-off threshold (Figure 1). The calculations were based on the incubation period of SARS-CoV-2 (Table 2), length of stay distribution of non-COVID-19 patients and assumed that all infections led to a symptom onset: hence it is the proportion of hospital-acquired infected individuals that will ever have symptoms and are identified (Supplementary 5). Uncertainty was included by sampling from parameter distributions (Table 2, Supplementary 10).

We did not account for misclassification of “community-acquired” as “hospital-acquired” as we estimated that fewer than 1% of inpatients with symptom onset 5 or more days after admission were latently infected when admitted i.e., hospital-onset, community-acquired (Table 1, Supplementary 3). Hence, our definition of “misclassified” only considers those “hospital-acquired” infections misclassified as “community-acquired”.

#### d. Reclassifying community-acquired COVID-19 cases as hospital-acquired

The number of patients with unidentified hospital-acquired infections was calculated by multiplying the number of identified hospital-acquired SARS-CoV-2 infections by the inverse of the proportion that were estimated to be identified (Figure 2). To determine the contribution of these unidentified hospital-acquired infections to the hospital burden of cases of COVID-19, we simulated their return as a COVID-19 hospital admission: we estimated the entire disease progression trajectory for each unidentified “missed” hospital-acquired infection by sampling from known natural history distributions (Figure 2) to determine how many may return to hospital and be misclassified “community-acquired” infection.

For each patient estimated to have had an unidentified “missed” hospital-acquired infection, we sampled a time from infection to discharge using the length of stay distribution of non-COVID patients (Supplementary 8), and assumed a date of discharge of 5 days before the detection date of the associated identified COVID-19 case (Figure 2c). This corresponds to the difference in the average length of stay of identified SARS-CoV-2 positive cases (~7 days) and those thought to be SARS-CoV-2 negative (~2 days) in SUS. In a sensitivity analysis, we explored the impact of this parameter by setting it to one day. From this date of discharge, we estimated the proportion of these unidentified “missed” infections expected to return as a hospitalised COVID-19 case as well as the timing of their return. The proportion expected to return varied for each simulation (Figure 2, Supplementary 6). Recalling exact dates of symptom onset is hard, hence we used a scenario analysis (scenarios 1-3) to explore three different distributions for the symptom onset to hospitalisation parameter (Table 2, Supplementary 7).

#### e. Hospital-linked cases

We defined a “hospital-linked infection” as an infection that occurred in the community but was caused by a patient that was estimated to have had an unidentified “missed” hospital-acquired infection. This time series of community infections was calculated by estimating four generations of onwards infection under varying assumptions about the reproduction number (Supplementary 6). This is approximately the number of infections caused within one month after discharge (~6.7 day serial interval, Supplementary 6).

We explored three reproduction number values: 1) a constant value of 0.8, 2) a constant value of 1.2 both with a range generated as +/− 5% of the constant value, and 3) a time-varying estimate “Rt” for which we used upper/lower bounds for the 50% credible interval from a publicly available repository (22) (Supplementary 9).

## Results

### Identified and classified hospital-acquired cases

In CO-CIN, using a symptom onset-based definition, we found 7% (n = 65,028) of COVID-19 cases in acute English Trusts were identified and classified as a hospital-acquired infection (having a symptom onset more than 7 days after admission and before discharge) before 31st July 2020. By adjusting for enrolment in CO-CIN (Figure 2b), we estimated that with this same cut-off there were 6,640 “hospital-onset, hospital-acquired” identified cases across acute English Trusts up to the 31st July 2020.

### Proportion of infections identified

**Figure 3:**
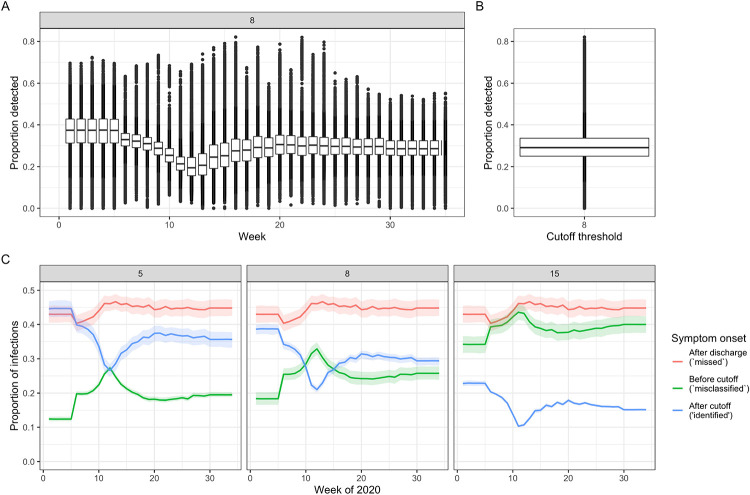
Proportion of symptomatic hospital-acquired infections identified, given by week (A) and over all weeks (B) at a 7 day cut-off, for all acute English Trusts. Each datapoint is the value from a single Trust for each of 200 samples. The boxplot highlights the median and 25th-75th quantile. (C) For England (the aggregate setting) the proportion of patients with hospital acquired infections split by those that are identified (blue) due to a symptom onset starting at a set number of days from admission (grey box) and before discharge, and those unidentified with symptom onset after discharge (“missed”, red) or before the cut-off (“misclassified”, green). The coloured lines represent the mean, and the shaded areas the 95% percentiles over the 200 samples.

We estimated 30% (20-41%, range across weeks and sampling, Supplementary 10) of symptomatic hospital-acquired infections (using a 7 day cut-off) were identified using a symptom onset based definition for England. Across all acute English Trusts the range was 0-82% (Figure 3). The proportion identified decreased with increasing cut-off day from admission (Figure 3c). The estimates are highly sensitive to LoS distributions (Supplementary 2). These results imply that for every single identified hospital-acquired SARS-CoV-2 infection (using a 7 day cut-off) there were, on average, two unidentified symptomatic hospital-acquired infections (Figures 1&2).

### Contribution of missed infections

We estimated that across England, 20,000 (mean; 95% range over 200 simulations to nearest 100:19,200, 21,100) hospital-acquired infections were unidentified from acute Trusts if a 7 day symptom-based cut-off was used to identify hospital-acquired cases. The majority of patients with unidentified hospital-acquired infections were not identified due to the discharge of the infected patient prior to symptom onset (“missed”) (Figure 1 and 3c): 12,300 (11,400, 13,400) in total.

A proportion of the patients with unidentified hospital-acquired infections with a symptom onset after discharge returned as hospitalised cases and were misclassified: we found 1,500 (1,200, 1,900) or 2.1% (1.7%, 2.6%) of cases originally classified as “community-onset, community-acquired” should have been classified as “community-onset, hospital-acquired” for a 7 day cut-off.

We found that there could have been 47,400 (mean; 95% range over 600 simulations: 45,000, 50,000 for the time-varying *R* value) hospital-linked infections of individuals in the community, acquired from patients with “missed” hospital-acquired infections during the first wave. We estimated that these hospital-linked infections would result in 1,600 (1,600, 1,700) “community-onset, hospital-linked” cases with a 7 day cut-off. The values are reduced by one-third with an *R* constant at 0.8 (Supplementary 11). These contribute 2.3% (2.1%, 2.4%) of “community-onset, community-acquired” cases over the first wave with a 7 day cut-off and under both scenario 1 or 2 (Supplementary 11).

**Figure 4:**
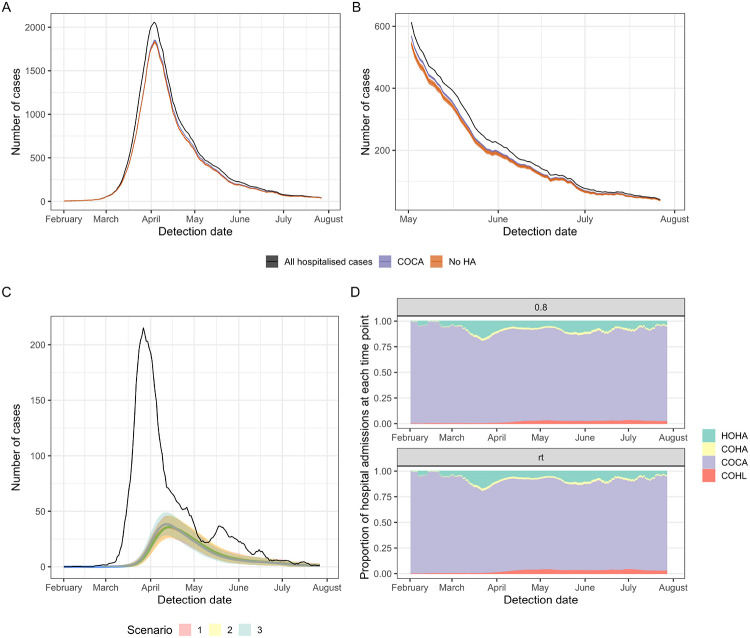
(A) Total COVID-19 admissions with model adjusted definitions from “community-onset, community-acquired” (COCA) for Scenario 1 for the whole study period (January - 31st July 2020) and (B) for the end of the study period (May - 31st July 2020). The counterfactual of no hospital transmission (“No HA”, orange) is compared to the adjusted model estimate of COCA (purple) and total admissions (black) for a time-varying R estimate. (C) The number of hospital-onset, hospital-acquired (HOHA) cases (black) is similar in magnitude to the number of community-onset hospital-linked (coloured lines, COHL) under the three scenarios for hospital admission after symptom onset. (D) The proportion of all hospital admissions in England that were estimated to be HOHA (green), community-onset, hospital-acquired (COHA, yellow), COCA (purple) and COHL (red) under two example R values (constant: 0.8 and time-varying “rt”) and Scenario 1. All outputs take a threshold cut-off value for defining hospital-acquired as a symptom onset more than 7 days from admission. All outputs are the rolling 7-day mean for the mean over 200 simulations with 5-95% ranges in shaded areas in (C).

This contribution of community-linked infections to hospital admissions with COVID-19 varied depending on the timing of hospital admission post symptom onset (captured here by Scenarios 1-3, Table 2, Figure 4). The proportion of COVID-19 hospital admissions due to hospital-transmission was greatest when total case numbers first declined (peak in COHL in Figure 4D at ~4% in late April).

The number of unidentified hospital-acquired infections and hence reclassification levels increased or decreased under a 14 or 4 day cut-off respectively (Supplementary 11).

### Contribution of hospital settings to cases, infections and onward transmission

**Figure 5:**
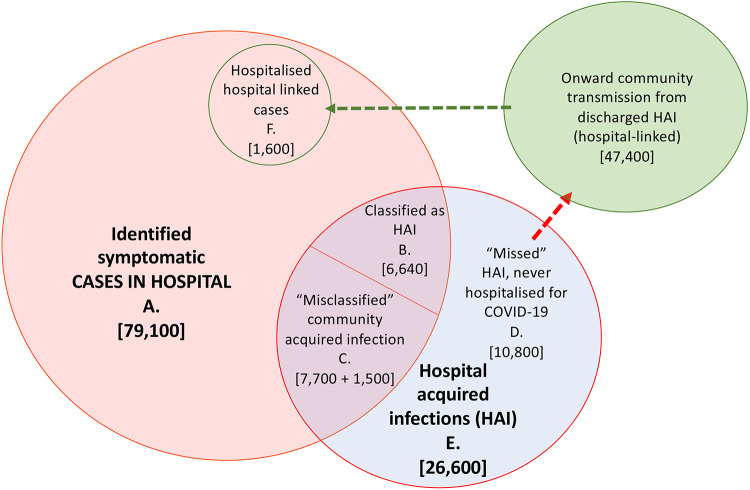
Summary figure of estimated values for patients with hospital-acquired symptomatic infections and onward community transmission with a 7 day cut-off for symptom onset after admission and prior to discharge for defining a patient with hospital-acquired infection. Note here that the “misclassified” (C) includes those “missed” unidentified infections that return to hospital later as a hospitalised COVID-19 case (1,500 “community-onset, hospital-acquired” cases).

To summarise, using a 7 day cut-off, we estimated that there have been a total of 26,600 (mean, 95% range over 200 simulations: 25,900, 27,700) symptomatic hospital-acquired SARS-CoV-2 infections in acute English Trusts (E, Figure 5) prior to August 2020. Of these, a total of 15,900 (15,200, 16,400) infections correspond to patients with COVID-19 that were identified as symptomatic cases in hospitals (B+C, Figure 5): as such only 60% of symptomatic hospital-acquired infections were identified. Over the whole first wave, we estimated that 15% (14.1%, 15.8%) of cases originally classified as community-acquired were hospital-acquired or hospital-linked ((C + F) / (A − B), Figure 5).

The estimated percentage of identified COVID-19 cases in hospitals that were hospital-acquired is then 20.1% (19.2%, 20.7%) ((B + C)/ A, Fig. 5). Accounting for onward transmission from unidentified “missed” hospital-acquired infections, we estimated that 22.1% (21.2%, 22.9%) of hospitalised COVID-19 cases were hospital-acquired or hospital-linked ((B + C + F)/A, Figure 5) using the median time-varying *R* value.

If 20.1% of COVID-19 cases identified in hospitals were hospital-acquired then, assuming that 3% of symptomatic cases were hospitalised, we estimated that hospital-acquired infections likely contributed to fewer than 1% of infections of the overall English epidemic of COVID-19 in wave 1.

Assuming similar levels of hospital transmission in non-acute English trusts suggests approximately 31,100 (30,300, 32,400) symptomatic infections could have been caused in total by symptomatic hospital-acquired transmission in England.

### Trust-level and Sensitivity analysis

When aggregated, the results from the analysis on an individual Trust-level predicted a slightly higher proportion of cases to be hospital-acquired (25% vs 20%) (Supplementary 12). Varying the day of discharge of the unidentified “missed” infections had little impact on total case numbers, but did affect hospital-linked cases (Supplementary 11).

## Discussion

We estimated that before 31st July 2020 20.1% (19.2%, 20.7%) of identified COVID-19 cases in hospitals were likely to have been hospital-acquired infections and that within-hospital transmission likely contributed directly to 26,600 (mean, 95% range over 200 simulations: 25,900, 27,700) symptomatic infections, and a further 47,400 (45,000, 50,000) hospital-linked infections. These results are based on a 7 day cut-off for symptom onset from admission and prior to discharge for defining an identified hospital-acquired case.

Despite these levels of infection, we estimated hospital transmission to patients caused fewer than 1% of all infections in England in the first wave (prior to 31st July 2020). To some extent this reflects effective infection prevention within hospital settings with over 4 million non-COVID-19 patients being cared for in hospital settings during this period. However, the high proportion of hospital cases that were due to hospital-acquired infections is worrying as these are the most vulnerable members of our society and hence may have the most severe consequences. In addition, we did not account for the substantial proportion of asymptomatic infections in our analysis and thus, the impact of hospital transmission on the community epidemic is likely an underestimate.(13)

This is the first study to estimate the total number of symptomatic hospital-acquired infections (not just the percentage of known cases that are hospital-acquired) and their wider contribution to community transmission prior to 31st July 2020. In particular, we found that the contribution of hospital-acquired infections to the epidemic likely varied over time, increasing in importance as community infections initially dropped, emphasising the need to determine where most infections are occurring at any one time during an epidemic.

Our results show that relying on symptom onset in inpatients as a detection method for hospital-acquired SARS-CoV-2 will miss a substantial proportion (> 60%) of symptomatic hospital-acquired infections. This depends on the length of stay for non-COVID admissions but suggests that in many settings estimates of the number of infections due to transmissions in hospital settings will be substantial underestimates. For example, Read et al (2021) acknowledged that the estimated proportion of nosocomial infections during the first epidemic wave of COVID-19 in the UK that was based on symptom onset data, is likely to be higher if accounted for unidentified cases (23). This is particularly relevant for low-resource settings with short lengths of stay for non-COVID patients and which rely on symptom onset screening for SARS-CoV-2 infection.

An alternative detection method is routine testing of patients, which will confirm symptomatic as well as detect pre-symptomatic and asymptomatic SARS-CoV-2 infections. However, even with screening on admission, symptomatic or not, and retesting 3 days after admission, a portion of infections will likely not be detected during inpatient stays due to short lengths of stay. Our estimates of the proportion of hospital cases that are due to hospital-acquired infection are higher than those from England wide studies (8,23) and those from single hospital settings in the UK (3,9,24-26), as we estimate all hospital-acquired infections whether identified or not during their hospital stay. Our estimates of all infections are similar to previous modelling work using an SEIR model which estimates that nosocomial transmission was responsible for 20% (IQR 14.4, 27.1%) of infections in inpatients.(27)

Our work implies that it may be effective to screen patients upon hospital discharge to detect infection, or to quarantine hospital patients on discharge to prevent ongoing community transmission: we estimate this would detect approximately 40% of hospital-acquired infections that would become symptomatic (that would otherwise be “missed” in Figure 3c). Hence, depending on the test sensitivity by time from infection, up to 70% of hospital-acquired infections could be detected. The onward community transmission from these infections may be especially important as community prevalence of SARS-CoV-2 infection decreases.

Currently, much more routine screening and testing is implemented in English hospitals contributing to the detection of infections prior to symptom onset or discharge.(28) However, screening will need to be conducted with high frequency to avoid missing those infected prior to discharge, or to screen on, and for several days after, discharge. Our work is directly linked to the situation prior to August 2020 where little routine testing was in place and would be affected substantially by the new pandemic situation with new variants and vaccination. However, our conclusion that symptomatic screening of inpatients has limited efficacy in detecting nosocomial transmission is still highly relevant to support the need for ongoing regular screening of asymptomatic hospital patients and to emphasize potential missing infections.

Further work is needed to determine the precise risk of returning as a hospital case for those infected in hospitals. If our values (10-15%) are found to be conservative, then this percentage could increase substantially. If it were found to be higher, reflecting the poorer health of hospitalised patients and hence potentially increased susceptibility, then the proportion of hospital cases that are hospital-acquired could increase to 30-40%.

The interpretation of our results is limited by several simplifications. Firstly, we did not explicitly capture disease and hospital attendance variation by age. Future work could stratify our estimates to account for an older and more vulnerable hospital population. Secondly, we likely underestimated the total number of hospital-acquired infections as we modelled only those that progress to symptoms. While a non-negligible proportion of SARS-CoV-2 infections is likely to be asymptomatic (13), hospital-acquired infections were defined using the date of symptom onset in the UK. In addition, (a proportion of) symptomatic infections require medical care and therefore directly contribute to the hospital burden. We, thus, focussed on estimating the magnitude of under detection of these symptomatic hospital-acquired infections and their wider impact on community transmission. Thirdly, we assumed a fixed number of four generations for onward transmission in the community, and did not account for infections in healthcare workers, nor in the setting to which hospitalised patients were discharged to, such as long-term care facilities. The impact of onward transmission from hospital-acquired infections may be underestimated in this work since these settings may have high levels and large heterogeneity in onward transmission or overestimated if four generations is longer than the average chain from recently hospitalised individuals. Fourthly, we assumed that equal levels of infection control policies were in place in all NHS Trusts during this time period as we had no data to inform variation. Moreover, some of the “missed” cases may have been detected by community screening although there was little in place in England in this time (prior to August 2020). Finally, identification of hospital infection using CO-CIN relied on symptom onset date, which may be unreliably recorded potentially leading to bias in the patient population. While we cannot assess the biases, it is reasonable to expect that symptoms were recorded well in a clinical setting, and frequently (~65,000 patients included). An alternative definition of hospital-acquired infection reliant on the date of first positive swab would have its own limitations: patients could enter with symptoms and not test positive until more than a week into their stay.(24)

## Conclusions

Due to the delay from infection to symptom onset, hospital-acquired transmission of SARS-CoV-2 may be missed under common definitions of a hospital-acquired infection. We estimated that nearly 20% of symptomatic COVID-19 patients in hospitals in England in the first wave acquired their infection in hospital settings. Whilst this is likely to have contributed little to the overall number of infections in England, the vulnerability of the hospital community means that this is an important area for further focus. Increased awareness and testing, especially of patients on discharge, as is now commonly in place, is needed to prevent hospitals becoming vehicles for SARS-CoV-2 transmission.

## Supplementary Material

Supplement 1

## Figures and Tables

**Table 1:** Case definitions. Terms are distinguished between surveillance definitions and quantities estimated in the analysis. Additional definitions are given in Supplementary 1.

	Term	Acronym	Classification	Explanation
Surveillance	hospital-onset, hospital-acquired case	HOHA	An individual hospitalised with COVID-19 with symptom onset after a defined cut-off of days from admission and prior to discharge	An individual identified with COVID-19 in a hospital that was presumed to be infected with SARS-CoV-2 in the hospital.
Surveillance	community-onset, community-acquired case	COCA	A hospitalised COVID-19 case with a symptom onset before a defined cut-off of days from admission and prior to discharge	An individual with identified COVID-19 in the hospital or community that was presumed to be infected with SARS-CoV-2 in the community.
Surveillance	Cut-off days for definition of hospital-acquired infection (in identified cases)		7 days (4 or 14 used in sensitivity analysis)	If symptoms onset occurs after this number of days from admission but before discharge then the case is identified as hospital-acquired.
To be estimated	unidentified hospital-acquired infection	“missed”	A person infected with SARS-CoV-2 during a hospital stay but not identified as symptom onset was after the patient was discharged	Our model estimates how many patients with hospital-acquired infections would be unidentified by using a definition of hospital-acquired that relies on symptom onset prior to discharge. We do not consider asymptomatic infections. We did not consider community-acquired infections “misclassified” as hospital-acquired as the percentage is very small after only a few days from admission (Supplementary 3, see HOCA below).
		“misclassified”	A person infected with SARS-CoV-2 during a hospital stay but not identified as symptom onset was before the defined cut-off	
To be estimated	Total number of patients with hospital-acquired infections		A person infected with SARS-CoV-2 during a hospital stay	The combined total of identified (those with symptom onset after a defined cutoff) and unidentified infections (“missed” and “missclassified”)
To be estimated	community-onset, hospital-acquired case	COHA	A hospitalised community-onset COVID-19 case that has a community-acquired classification but was actually a unidentified hospital-acquired infection.	Our model prediction of how many unidentified hospital-acquired infections would return as a hospitalised COVID-19 case. These need to be re-classified as hospital-not community-acquired.
To be estimated	community-onset, hospital-linked case	COHL	A hospitalised community-onset COVID-19 case that was infected in a chain of four generations of transmission that started with an unidentified hospital-acquired infection.	Our model prediction of the contribution of unidentified hospital infections to onward community transmission approximately one month after discharge.
Minimal	Hospital-onset, community-acquired case	HOCA	Symptoms after the cutoff for defining hospital-acquired, but infection was in the community.	We estimate that less than 1% of those with symptom onset more than 5 days from admission would have a community-acquired infection (Supplementary 3)

**Table 2:** Parameters values used in the model. See Supplementary 6 for more details.

Definition		Values/Distributions	Refs
Proportion of individuals with unidentified hospital-acquired infections that will be subsequently admitted to hospital with COVID-19		unif(range = 0.1-0.15)	[40-42])
Proportion of community infections that will be hospitalised cases of COVID-19		norm(0.035, 0.0005)	[40]
Time to symptom onset from infection (incubation distribution)	mean distribution	lognormal(mean = 1.62, sd = 0.4)	[22]
	standard deviation in estimates of mean and standard deviation	0.064	
		0.0691	
Time to hospitalisation from symptom onset	Scenario 1 (baseline):	Lognormal (mean = 1.66, sd = 0.89)	Supplementary 7 [32,35,43]
	Scenario 2:	gamma(shape = 7, scale = 1)	
	Scenario 3:	Lognormal (mean = 1.44, sd = 0.72)	
Time from infection to hospitalisation	Sum of means of infection to symptom onset and symptom onset to hospitalisation = 5.1 + 7 = 12.1 days		
Average number of secondary infections from one infected individual in the community (*R*)	0.8, 1.2 and “rt”		[39], Supplementary 9
Time period over which an infected individual is infectious	gamma(shape = 4, scale = 0.875)		[43]
Number of days before associated identified hospital-acquired case detection that a patient with a unidentified “missed” hospital-acquired infection is discharged from hospital	5 or 1		Assumptions

## Abbreviations

CO-CIN: ISARIC4C UK COVID-19 Clinical Information Network
COCA: community-onset, community-acquired infection
COHA: community-onset, hospital-acquired infection
COHL: community-onset, hospital-linked infection
COVID-19: Coronavirus Disease 2019
HCW: Healthcare worker
HOCA: Hospital-onset, community-acquired infection
HOHA: hospital-onset, hospital-acquired infection
NHS: National Health Service (the public health service in Britain that provides medical treatment and is paid for by taxes)
UK: United Kingdom
SARS-CoV-2: Severe Acute Respiratory Syndrome Coronavirus-2
SUS: Secondary Uses Service (collection of health care data required by hospitals and used for planning health care, supporting payments, commissioning policy development and research)
PCR: Polymerase Chain Reaction
